# Studies on *Escherichia coli *HflKC suggest the presence of an unidentified λ factor that influences the lysis-lysogeny switch

**DOI:** 10.1186/1471-2180-11-34

**Published:** 2011-02-17

**Authors:** Kaustav Bandyopadhyay, Pabitra K Parua, Ajit B Datta, Pradeep Parrack

**Affiliations:** 1 Department of Biochemistry, Bose Institute, P 1/12, C.I.T. Scheme VIIM, Kolkata 700 054, India; 2 Department of Biochemistry, University of Washington, 1705 NE Pacific St. HSB J-405, Box 357350 Seattle, WA 98195-7350, USA; 3 Department of Biophysics & Biophysical Chemistry and Howard Hughes Medical Institute, Johns Hopkins University School of Medicine, Baltimore, MD 21205, USA

## Abstract

**Background:**

The lysis-lysogeny decision in the temperate coliphage λ is influenced by a number of phage proteins (CII and CIII) as well as host factors, viz. *Escherichia coli *HflB, HflKC and HflD. Prominent among these are the transcription factor CII and HflB, an ATP-dependent protease that degrades CII. Stabilization of CII promotes lysogeny, while its destabilization induces the lytic mode of development. All other factors that influence the lytic/lysogenic decision are known to act by their effects on the stability of CII. Deletion of *hflKC *has no effect on the stability of CII. However, when λ infects *ΔhflKC *cells, turbid plaques are produced, indicating stabilization of CII under these conditions.

**Results:**

We find that CII is stabilized in *ΔhflKC *cells even without infection by λ, if CIII is present. Nevertheless, we also obtained turbid plaques when a *ΔhflKC *host was infected by a *cIII*-defective phage (*λcIII*^*67*^). This observation raises a fundamental question: does lysogeny necessarily correlate with the stabilization of CII? Our experiments indicate that CII is indeed stabilized under these conditions, implying that stabilization of CII is possible in *ΔhflKC *cells even in the absence of CIII, leading to lysogeny.

**Conclusion:**

We propose that a yet unidentified CII-stabilizing factor in λ may influence the lysis-lysogeny decision in *ΔhflKC *cells.

## Background

After it infects host *E. coli *cells, bacteriophage λ follows either of two fates, lytic or lysogenic. How the virus decides which pathway to follow after infection depends upon a complex genetic circuit. An increase in the number of infecting phages converts the decision making process from a deterministic to a stochastic one, with the cell fate depending on the number of phages deciding in favour of lysogeny [1,2]. There are phage coded proteins and transcription factors [3-5] dedicated for this decision making process, but host factors are also involved [6-9]. Mutations in the *cI, cII *and *cIII *genes of λ [10] enhances the lytic frequency (leading to clear plaque formation, hence the names) and therefore the products of these genes were thought to be responsible for the establishment of lysogeny. CII, the key tetrameric transcription factor for lysogenic establishment, is a very unstable protein [7,11,12] and its presence in sufficient amounts is crucial for the lysogenic choice [13-15]. Other factors such as λCIII and the host hfl proteins that influence the lysis-lysogeny switching affect the stability of CII in one way or the other. λCIII promotes lysogeny by acting as a general inhibitor of *E. coli *HflB that degrades CII [16].

Mutations in the host *hfl *loci cause an infecting λ particle to follow the lysogenic mode. These genes therefore encode factors that somehow destabilize CII. Primarily from mutational studies, two such loci, *hflA *and *hflB*, were initially identified. The product of the latter gene, HflB, is an ATP-dependent metalloprotease known as a 'quality control' protease that removes misfolded proteins produced due to rapid translation during good nutrient conditions [17,18]. CII is also a substrate of HflB [7] and thus acts as a sensor for cellular nutrient conditions of the host. Rapid degradation of CII in cells growing in rich media thus favors the lytic development [13,14]. The *hflA *locus consists of the genes *hflX*, *hflK *and *hflC *that are under the control of the same promoter [19-22]. Of these, *hflX *has been demonstrated to have no role in lambda lysogeny [23]. The products of the other two, HflK and HflC, are tightly associated with each other and copurify as the 'HflKC' complex, which was earlier thought to be a protease [24]. Subsequently, HflKC was found only to act as a 'modulator' of HflB by forming a complex with the latter [25-27]. The only other known *E. coli *factor in this process, HflD [9], has been shown to inhibit CII-mediated activation of transcription by impairing the DNA-binding ability of CII [28].

HflKC antagonizes the action of HflB towards the membrane associated substrates of the latter [18,25]. The behavior of HflKC with respect to the cytosolic substrates of HflB (such as λCII), however, remains unclear. Likewise, the role of HflKC in the lysis-lysogeny decision of λ is not well understood. Though an 'hfl' protein, mutations in whose gene(s) causes an increase in the lysogenic frequency of λ [6], the deletion of these genes has little effect on the *in vivo *stability of exogenous CII [26]. CII expressed from a plasmid is found to be stabilized in an *hflKC-*deleted cell, only if the host is simultaneously infected with a lambda phage [26]. On the other hand, *E. coli *cells overexpressing HflKC exhibit an enhanced frequency of lysogenization [26]. These results lead to a paradox: lysogeny increases both upon deletion or overexpression of HflKC. Therefore, the role of HflKC in the λ lysis-lysogeny switch merits further investigations.

## Methods

### Plasmids, bacterial strains and phages

Plasmid pQKC was constructed by PCR cloning of the *hflK *and *hflC *ORFs (not fused, because the genomic region between these two contains the stop codon for *hflK *and the RBS for *hflC*) between the *Bam*HI and *Sal*I sites of pQE30 (purchased from Qiagen, contains the phage T_5 _promoter under the control of a Lac operator). Construction of pKP219 (which contains the *cII *gene under the lac promoter LacP and a P15A replication origin) has been described earlier [28]. Plasmid pC2C3 (containing the *cII *and *cIII *genes) was constructed in three steps. First, the *Nde*I-*Bam*HI fragment of pAB905 containing the *cIII *gene [29] was cloned into pKP07 [28] and was named pLaCIII (containing the *cIII *gene under LacP). Then the *Bgl*II-*Xho*I fragment of pLaCIII (i.e. the cIII gene along with the LacP) was cloned into the compatible *BamH*I-*Xho*I sites of pKP106 (which already contained the cII gene under LacP) [28]. The resulting plasmid was named pLaC2C3. In the final step the *Bam*HI-*Bgl*II fragment of pLaC2C3 (containing both *cII *and *cIII *under individual LacP promoters) was cloned into the linearized arm of pK109 (having a P15A origin of replication) [30] at the *Bgl*II site.

For wild type *E. coli*, the strain MG1655 (*F*^- ^*λ*^- ^*ilvG rfb-*50 *rph-1*) was used. The strain AK990 [26] (*ΔhflKC:: Kan*) served as cells with mutant *hflKC*.

The phage strain λ*cIII*^67 ^was used as the CIII-defective phage. In this strain, a G to T mutation in the 23rd nucleotide of the *cIII *ORF leads to an alternative structure of the cIII mRNA that is incapable of translation [31]. This is one of the most effective cIII mutants [32] and has been used as cIII^- ^by many workers.

### Purification of proteins

For the purification of the HflKC complex, XL1Blue cells carrying pQKC was used and 100 μg/ml of ampicillin was used for selection. 7.5 ml of the overnight saturated culture was inoculated into 750 ml of fresh M9 medium with the appropriate antibiotic and allowed to grow on a 37°C shaker incubator till the culture O.D. (at 600 nm) was 0.4-0.5. The culture was then cooled to 18°C and induced by 500 μM IPTG, followed by further growth at 18°C with constant shaking (at 100 rpm) for 20 hours. After induction, bacterial cells were recovered by centrifugation at 3000 g for 10 minutes in Sorvall RC5C, using an SA600 rotor, at 4°C. The medium was decanted out and the pellet was washed with 0.9% NaCl and dissolved in 20 ml of lysis buffer (20 mM TRIS-HCl, pH 8.0, 100 mM KCl, 10% glycerol, 5 mM imidazole, 0.5% NP40, bacterial protease inhibitor cocktail (MBI Fermentas) and 200 μg/ml lysozyme). Cells were then lysed by sonication with 5 pulses (at a pulse rate of 10 mV/30 seconds), followed by centrifugation at 26000 g for 30 minutes at 4°C. The supernatant was collected into a fresh tube and loaded on to a Ni^+2^-NTA column, pre-equilibrated with the lysis buffer. After loading, the column was washed with wash buffer (20 mM TRIS-HCl, pH 8.0, 600 mM KCl, 10% glycerol, 15 mM imidazole). Proteins were eluted from the column using the elution buffer (20 mM TRIS-HCl, pH 8.0, 100 mM KCl, 10% glycerol, 0.1% NP40, 300 mM imidazole). Imidazole was removed by dialysis in 20 mM TRIS-HCl, pH 8.0, 100 mM KCl, 10% glycerol, 0.1% NP40).

Native CII [33] and GST-HflB [29] were purified as described earlier.

### *In vitro *proteolysis of CII

HflB mediated proteolysis of CII was carried out in buffer P (50 mM Tris-acetate, 100 mM NaCl, 5 mM MgCl_2_, 25 μM Zn-acetate, 1.4 mM β-ME; pH 7.2). ATP was added to a concentration of 5 mM in all the reaction mixtures. 8 μM of CII was taken with 1 μM of purified GST-HflB in a 30 μl reaction mix. The reactions were incubated at 37°C for the specified time intervals followed by the addition of SDS-PAGE loading buffer and heating in a boiling water bath for 5 minutes. The samples were analyzed on a 15% SDS-PAGE. The effect of HflKC on the proteolysis of CII was observed by the addition of His-HflKC (up to 2 μM) to GST-HflB prior to the addition of CII. The band corresponding to CII was quantitated by volume analysis (software used: Versadoc (Bio rad) Quantity-1) and used as the amount of CII remaining (expressed as the percentage of the amount of CII at zero time) after the specified time.

### *In vivo *proteolysis of CII

*In vivo *proteolysis of CII was carried out in *E. coli *MG1655 cells (having wild type HflB) transformed with pKP219 or pC2C3, both of which contained *cII *under Lac promoter. In addition, pC2C3 contained *cIII *under a second Lac promoter. Cells carrying pKP219 or pC2C3 were inoculated in 10 ml of LB medium supplemented with 50 μg/ml kanamycin. Expression of CII was induced by 1 mM IPTG after the O.D. of the culture (at 600 nm) had reached 0.6. The culture was further grown at 37°C for another 30 minutes, followed by the addition of 10 μg/ml spectinomycin to arrest further protein synthesis. Samples were taken out at regular intervals after spectinomycin addition, and immediately centrifuged to pellet the cells. 30 μl of sterile water and 8 μl of SDS gel loading dye were added to each sample, followed by immediate boiling and loading onto a 15% SDS-PAGE. The gel was transferred to a PVDF membrane (Pierce Biotech) and was blotted with anti-CII antibody. Each CII band was quantitated by volumetric analysis as described above.

The effect of overexpression of *hflKC *was observed by transformation of MG1655 cells by plasmid pQKC (plus pKP219 or pC2C3). The transformed cells were grown in the presence of both kanamycin and ampicillin. Promoters in both the plasmids are inducible with IPTG. The effect of deletion of *hflKC *was observed by transformation of AK9990 cells by pKP219 or pC2C3.

For measurement of the stability of CII under conditions of infection by λ*cIII*^67^, MG1655 or AK990 cells carrying pKP219 were grown in Luria broth supplemented with 0.4% maltose and were infected with the phage (at an MOI of 10 to ensure that all the cells were infected), 20 minutes after the addition of IPTG. Spectinomycin was added after another 25 minutes to ensure the entry of phage DNA and the expression of phage factors. Samples were then taken out at regular intervals and analyzed as described above.

### Assay of plaque morphology

The plaque morphology of λ*cIII*^67 ^was assayed in *E. coli *MG1655 (wild type), in MG1655 cells carrying pQKC, and in strain AK990 (*ΔhflKC::Kan*). Cells were grown up to an O.D. (at 600 nm) of 0.6 in Luria broth supplemented with 0.4% maltose, and were induced with 500 μM IPTG. A bacterial lawn was made by pouring 5 ml of soft top agar (0.5% Luria agar supplemented with 0.4% maltose) mixed with 300 μl of these cells onto a 2% Luria agar plate. Another 100 μl of the above liquid culture was infected with λ*cIII*^67 ^at an M.O.I. of 0.1. It was further incubated at 32°C for 10 minutes to allow adsorption of the phage. Appropriate dilutions were then plated onto the prepared bacterial lawn and the plates were incubated overnight at 32°C. The turbidity of plaques formed in AK990 cells or in cells overexpressing HflKC were compared with the clear plaques formed in wild type cells upon infection by λ*cIII*^67^.

## Results and Discussion

### Role of HflKC on the proteolysis of CII *in vivo*

**Figure 1:**
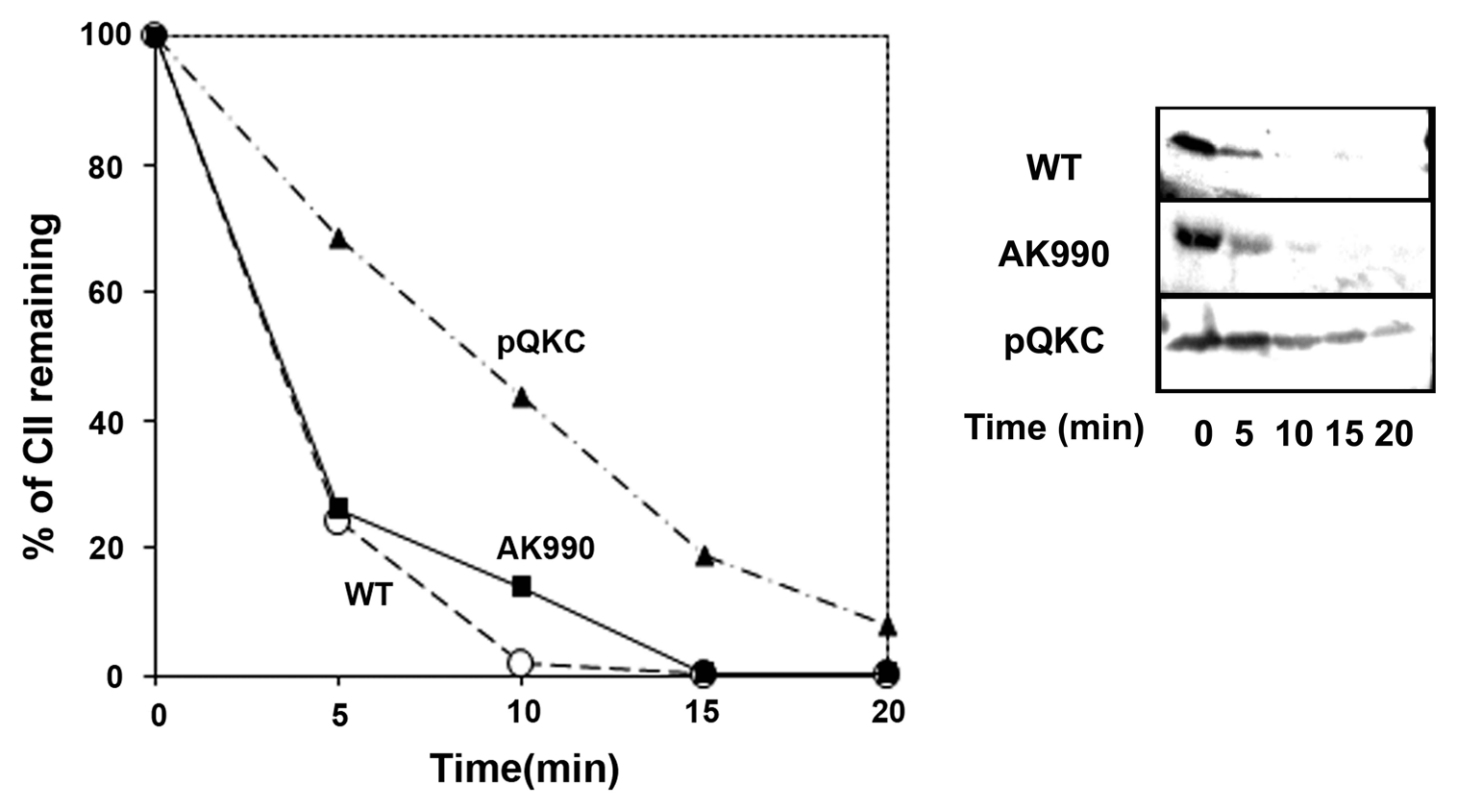
**Role of HflKC on *in vivo *proteolysis of CII**. Left panel shows the proteolytic pattern of exogenous CII (expressed from pKP219) in wild type cells (open circles), AK990 (Δ*hflKC*, squares) or wild type cells carrying plasmid pQKC (triangles). In each case, the expression of CII was induced with IPTG and translation was stopped 30 minutes later with spectinomycin. Relative amount of CII was measured after regular intervals (0, 5, 10, 15, 20 minutes) by western blotting followed by quantification using densitometric analysis. Corresponding western blots showing the stability of CII in different host strains are shown in the right panel.

*E. coli *HflKC inhibits the proteolysis of all the membranous substrates of HflB (e.g., SecY, YccA) [18]. However, the behaviour of HflKC toward λCII, a cytosolic substrate, is perplexing. The deletion of *hflKC *as well as its overexpression causes an increase in the lysogenic frequency of λ [26]. The *hflKC *genes were first identified as mutants that caused turbid plaques of λ on a bacterial lawn [6]. It is therefore expected that CII would be stabilized in an *hflKC-*deleted host cell. Kihara *et al. *[26], however, showed that the deletion of *hflKC *has little effect on the stability of CII cloned under an AraBAD promoter. We obtained similar results when the effect of *hflKC *deletion (strain AK990) on the stability of CII (cloned under *lac *promoter) was tested (Figure 1). Here we measured the stability of CII expressed from the plasmid pKP219 in wild type and in AK990 (Δ*hflKC*) cells. In both cases, CII was unstable. We also tested the effect of overexpression of HflKC from a second plasmid (pQKC), and found that in this case, CII expressed from pKP219 was stabilized (Figure 1). This data is consistent with *in vitro *results that showed that purified HflKC [26,34] inhibits the proteolysis of CII. The inhibitory activity is an intrinsic property of HflK and HflC, since HflK or HflC can individually inhibit the proteolysis of CII [34].

These results pose an intriguing question. Why does the deletion of an inhibitor of CII proteolysis promote lysogeny? One can think of the following possibilities: (i) A proper assembly of HflB that is necessary for its activity against cytosolic substrates, may require HflKC; or (ii) In the absence of HflKC, HflB is guided towards its membrane-associated substrates [26], indirectly stabilizing the cytosolic substrate CII. However, from *in vivo *proteolysis experiments we found that in AK990 cells (Δ*hflKC*), exogenous CII was not stabilized (Figure 1), confirming that HflB was active against CII even in the absence of *hflKC*. This result rules out both the possibilities mentioned above. It may be noted that similar results were also obtained by Kihara *et al *[26]. Therefore, an increase in lambda lysogeny upon overexpression of host HflKC [26] is not at all surprising, since HflKC inhibits the proteolysis of CII.

### Effect of increasing concentrations of HflKC on the proteolysis of CII *in vitro*

**Figure 2:**
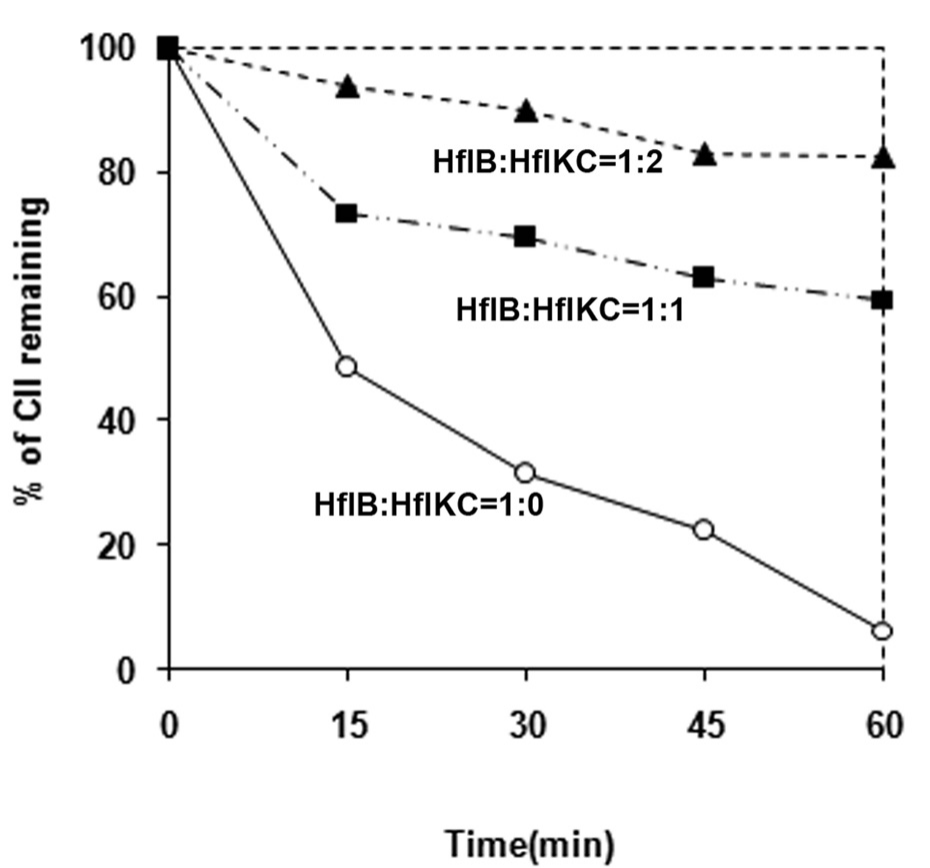
**Effect of varying concentrations of HflKC on *in vitro *proteolysis of CII**. CII (8 μM) was treated with GST-HflB (1 μM), in the presence of His-HflKC in various concentrations: 0 (open circles), 1 μM (squares) and 2 μM (triangles). Samples were taken out at various time points, run on a 15% SDS-PAGE, and the CII bands were quantitated by densitometry.

The paradoxical effect of an increase in the lysogenic frequency of λ upon deletion as well as overexpression of *hflKC *has been reported [26]. A possible reason behind this paradox could be that a critical molar ratio between HflB and HflKC, believed to be 1:1 in wild type cells [35], is necessary for a proper proteolysis of CII by HflB. Both the increase or decrease of HflKC would offset this critical ratio and could lead to a stabilization of CII, promoting lysogeny. To examine this possibility, we carried out the proteolysis of CII by HflB *in vitro*, in the presence of three different concentrations of HflKC (Figure 2). In the first case, when HflKC was absent (mimicking the deletion of HflKC), CII (8 μM) was rapidly cleaved by HflB. The rate of proteolysis was much slower when HflKC was added in a molar ratio of HflKC:HflB = 1:1. The proteolysis was inhibited further when HflKC was added in excess (HflKC:HflB = 2:1). If the above hypothesis was true, proteolysis of CII should have been maximum at a molar ratio of 1:1. Therefore we conclude that HflKC acts as a simple inhibitor of CII proteolysis and the stabilization of CII in the absence of HflKC may involve other factors.

### Role of HflKC on *in vivo *proteolysis of CII: the effect of CIII

Deletion of *hflKC *genes lead to an increase in the lysogenic frequency of λ. Nevertheless, the stability of cloned CII remained unaffected in Δ*hflKC *cells. An interesting phenomenon, however, was observed in Δ*hflKC *cells that were infected by λ. CII expressed from a plasmid was stabilized in these cells [26]. Thus it appears that some additional factors, supplied by the infecting phage, caused a stabilization of CII in the absence of HflKC. The only known phage factor that favors lysogeny by inhibiting the proteolysis of CII by HflB, is CIII [29,36]. We therefore tested the possible involvement of CIII as the λ factor responsible for the above result, *viz. *stabilization of CII in λ-infected Δ*hflKC *cells.

**Figure 3:**
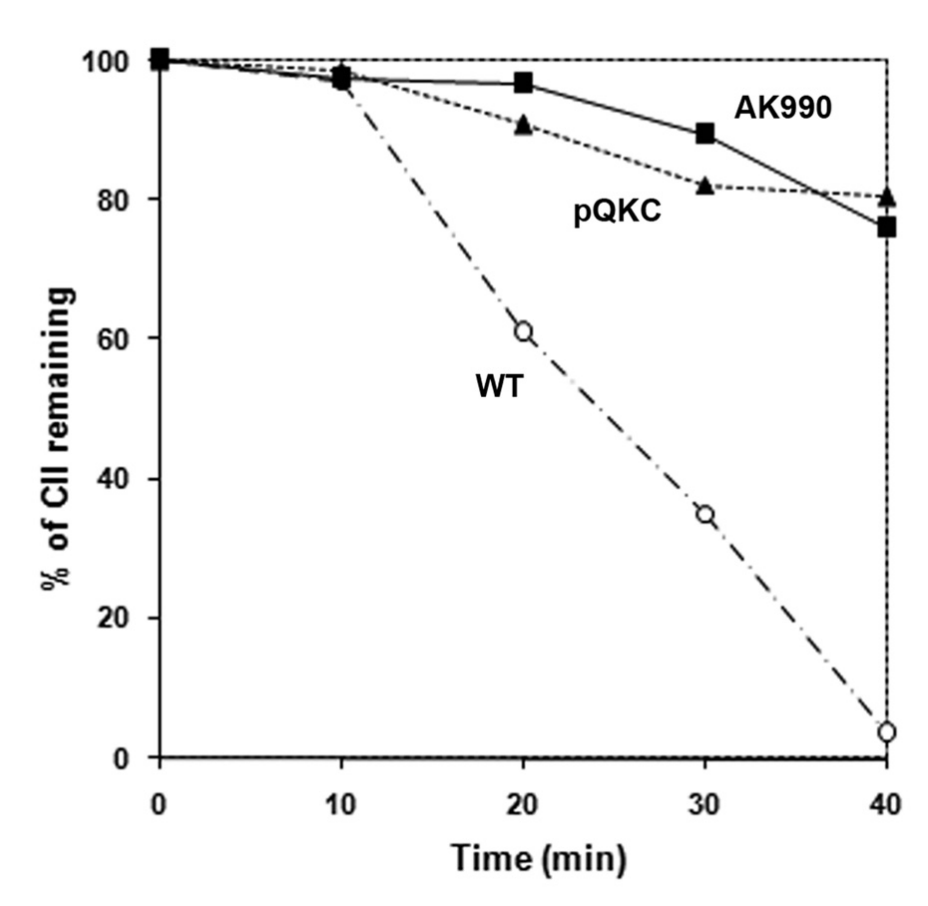
**Role of HflKC on *in vivo *proteolysis of CII in the presence of CIII**. Proteolytic pattern of exogenous CII (expressed from pC2C3) in wild type cells (open circles), AK990 (Δ*hflKC*, squares) or wild type cells carrying plasmid pQKC (triangles). Experimental conditions were similar to those used in Figure 1.

We sought to supply λCIII instead of the whole phage in an *hflKC-*deleted host and investigate its effect on the proteolysis of cloned CII. For this purpose, we cloned *cIII *in tandem behind *cII *in the same plasmid and inserted it in a host with deleted (AK990) or overexpressed *hflKC*. CII was indeed stabilized in these cells, even without simultaneous infection by λ (Figure 3). Therefore it appears that infection by λ stabilized CII in Δ*hflKC *cells because it supplied CIII.

CIII is a general inhibitor of CII proteolysis [29,36,37]. It is therefore expected that between a wild type strain alone and one that carries CIII, CII would exhibit a greater stability in the latter. A comparison of figures 1 and 3 (open circles) shows that this is indeed the case. Nonetheless, a greater stability of CII in Δ*hflKC *cells compared to wild type (both carrying the CIII-expressing plasmid) is surprising, since the absence of *hflKC *does not affect the stability of CII. CIII is itself a substrate of HflB [38]. If HflKC facilitated the proteolysis of CIII, the above effect could be explained by the preferential stabilization of CIII in Δ*hflKC *cells. However, there was no difference in the *in vitro *proteolysis of CIII by HflB in the presence or absence of purified HflKC (data not shown). Therefore the role of CIII in this paradoxical effect is indirect.

### Are there additional λ factors that influence the lysis-lysogeny decision?

If CIII was the only factor responsible for the stabilization of CII in Δ*hflKC *cells, infection with a *cIII*-defective phage would produce clear plaques in a Δ*hflKC *host. We tested this possibility by infecting both AK990 (Δ*hflKC*) cells and *hflKC*-overexpressing cells with lambda *cIII*^*67 *^[31,39]. Interestingly, turbid plaques were obtained in each case, unlike the clear plaques produced in wild type *E. coli *(Table 1). This result is really surprising as *cIII *^- ^phage always produces clear plaques. Since CIII and HflKC both inhibit the proteolysis of CII, it is also surprising that the absence of both leads to increased lysogeny.

**Table 1:** Plaque morphology upon infection with λ*cIII*^67^

Genotype of host *E. coli *cell	Plaque morphology
Wild Type	Clear
Wild Type + pQKC	Turbid
AK990 (Δ*hflKC::Kan*)	Turbid

**Figure 4:**
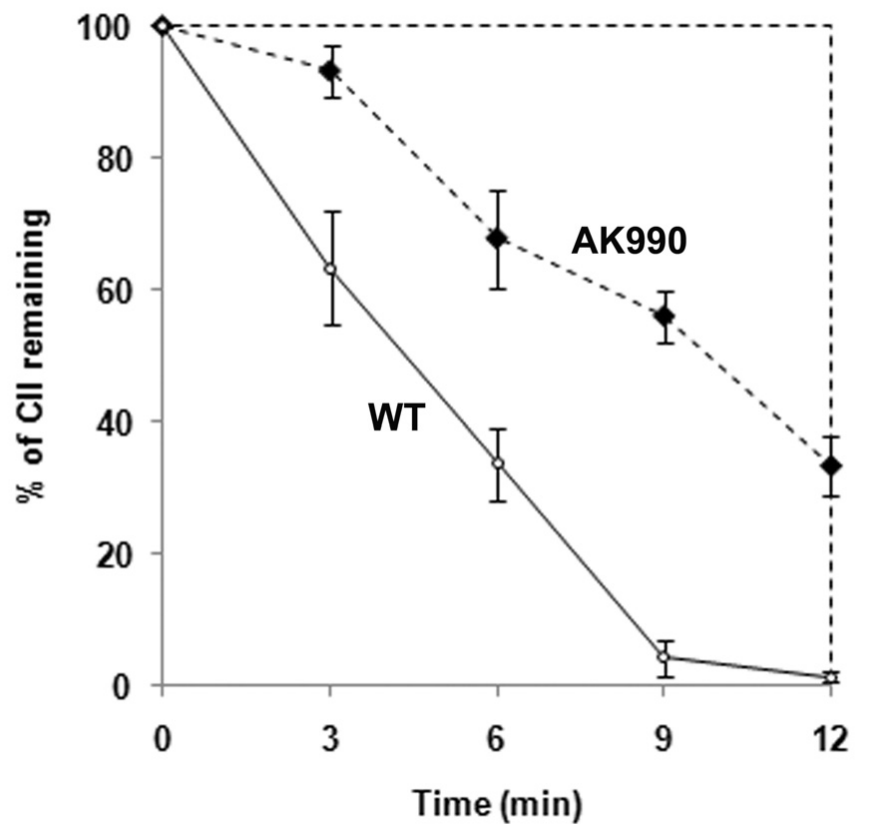
**Effect of infection by *cIII*-mutant lambda on *in vivo *proteolysis of CII**. The proteolysis of CII was visualized in wild type (open circles) or AK990 (diamonds) cells infected with λ*cIII*^*67*^. The expression of CII was induced with IPTG, and the cells were infected with the phage after 20 minutes. Protein synthesis was stopped 25 minutes later with spectinomycin. The relative amount of CII was measured at regular intervals by western blotting followed by quantification using densitometric analysis.

Is it then possible that enhancement of lysogeny can occur through a different mechanism that does not involve the stabilization of CII? Increase in lambda lysogeny is invariably linked to the stability of CII in all published reports to date. Can the two phenomena be delinked in some special case such as a Δ*hflKC *host? We tested this possibility by measuring the stability of cloned CII in wild type and Δ*hflKC *cells, both infected with λ*cIII*^*67*^. A greater stabilization of CII occurred in Δ*hflKC *cells (Figure 4). Therefore, an increase in the lysogenic frequency indeed requires the stabilization of CII.

**Figure 5:**
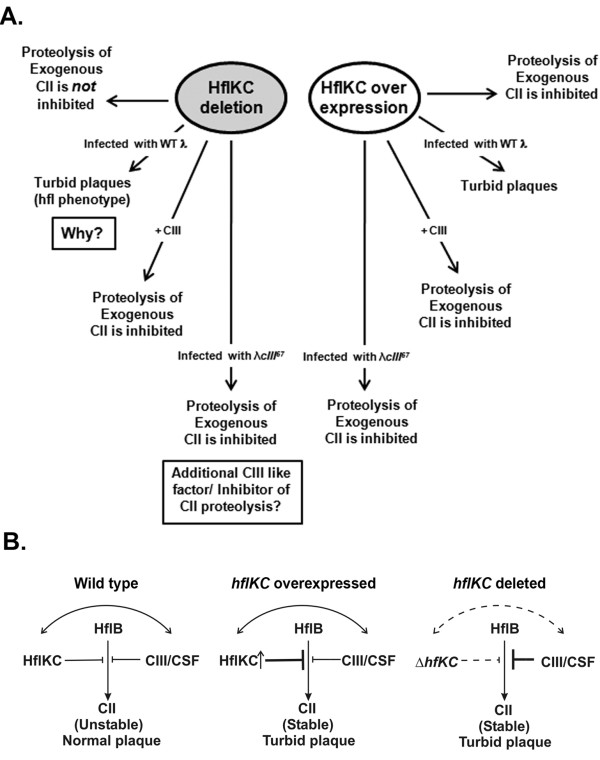
**The effect of deletion or overexpression of hflKC on λ lysogeny and on the stability of CII: A summary of results and possible mechanisms**. (A) A summary of results published previously as well as reported in this study is shown schematically. Some unanswered questions that remain are highlighted in the boxes. (B) Mechanisms for the stability of CII and the lysogenic outcome under various conditions are shown. HflB acts upon CII to digest CII, as indicated by the arrow. This digestion is inhibited by HflKC, by CIII or by the postulated CII-stabilizing factor CSF. The levels of inhibition are denoted by the lengths of the blunt lines. Possible crosstalk between HflKC and CIII or CSF are indicated by curved arrows. Dashed arrows denote lack of crosstalk. HflKC, CIII or CSF inhibits the digestion of  CII. In wild type E. coli cells, this inhibition is unable to sufficiently stabilize CII, leading to normal plaques (left panel). When HflKC is overexpressed, CII is stabilized better by the action of HflKC, and turbid plaques are produced (middle panel), while in Δ hflKC cells, CIII and/or CSF act better to stabilize CII, giving rise to turbid plaques (right panel).

This enhanced stabilization of CII is observed only under conditions of phage infection, even when CIII is nonfunctional. Therefore in addition to CIII, there could be another as yet unidentified factor in λ that increases the stability of CII and hence, promotes lysogeny (see Figure 5A). The presence of such a CII-stabilizing factor (CSF) can only be demonstrated in HflKC-deleted cells. Therefore, the activities of CSF and HflKC must have some connections (Figure 5B). Likewise, CIII and HflKC are likely to be connected as well. The different outcomes for deletion or overexpression of *hflKC *on lysogeny as well as on the stability of CII under various conditions are summarized in Figure 5A.

The unknown factor CSF could have been a non-protein factor (i.e, DNA) and lambda DNA would have been a good candidate for the same, since CII may be stabilized by binding to its cognate promoter. However, in our *in vivo *experiments, the plasmid pKP219 (used for the expression of exogenous CII) contained the promoter sequence P_E_, ruling out such a possibility.

Stabilization of CII in cells overexpressing *hflKC *is not surprising since HflKC is an inhibitor of CII-proteolysis. It is worthwhile to note that the effect of HflKC deletion is epistatic over the effect of *cIII *deletion, since even the absence of CIII cannot produce clear plaques in a Δ*hflKC *host. It is possible that CIII (and the hypothesized CIII-like factor CSF) works better in the absence of HflKC (Figure 5B). Therefore CII is better stabilized under these conditions and produces turbid plaques in *ΔhflKC *cells. *cI*, *cII *and *cIII *were first described as phage mutations which led to clear plaques in a wild type host. On the other hand, λ gives very turbid plaques in a Δ*hflKC *host. Our study thereby raises the possibility of finding novel phage mutations that would give clear plaques in an *hflKC*-deleted host.

## Conclusions

1. *E. coli *HflKC inhibits the proteolysis of λCII by HflB and hence the overexpression of the former results in an increase in the lysogenic frequency.

2. In the absence of HflKC, λCII is stabilized upon infection by cIII-defective λ, suggesting the presence of a yet unidentified phage factor CSF (CII-stabilizing factor).

## Authors' contributions

KB and PP designed the experiments, KB performed the experiments and analysed the results of the HflKC-based *in vitro *and *in vivo *experiments. PKP designed and constructed the vector pKP219 and designed the method to determine the stability of CII *in vivo*. ABD helped in designing experiments and drawing inferences from the experimental results. PP designed research and supervised all the work. KB and PP wrote the manuscript and all authors approved the final version.
